# Introduction to Special Issue, Aging and Public Health

**DOI:** 10.1093/geroni/igaa008

**Published:** 2020-03-18

**Authors:** Steven M Albert, Vicki A Freedman

**Affiliations:** 1 Department of Behavioral and Community Health Sciences, Graduate School of Public Health, University of Pittsburgh, Pennsylvania; 2 Institute for Social Research, University of Michigan, Ann Arbor

In developing this special issue of *Innovation in Aging*, we asked researchers to tackle a central question in aging and public health: “Will population aging be accompanied by a longer period of good health, a sustained sense of well-being, and extended periods of social engagement and productivity, or will it be associated with more illness, disability, and dependency?” (WHO, 2015; WHO and National Institute of Aging, 2011). WHO suggests a public health framework for healthy aging that stratifies older populations by functional trajectory and intrinsic capacity to identify three broad subpopulations: a group with high and stable capacity, a group with declining capacity, and a group with significant loss of capacity. The optimal mix of interventions will differ for each. For example, preventing chronic conditions (and ensuring early detection and control) is always a priority, but public health and aging efforts also need to promote compensation for lost capacities. In older adults with advanced chronic conditions, for whom prevention is no longer possible, removing barriers to participation may be as important as treatment.

Public health faces the challenge of designing, assessing, translating, and implementing programs that push interventions out to aging subpopulations that span this continuum of health and vulnerability. This effort requires population health surveillance as well as implementation of programs that utilize infrastructures on the ground, such as health departments, aging services providers, the Centers for Disease Control and Prevention (CDC), the Administration for Community Living/Administration on Aging, state units on aging, county human services, health systems, and national advocacy organizations.

How well did the papers in our issue meet this challenge? The eight papers that successfully completed peer review (out of more than 50 abstracts submitted) can be divided into three types. Papers focused on (i) making communities and service systems more age-friendly, (ii) population surveillance of vulnerable subgroups of the aging population, and (iii) programmatic innovations to address specific needs common among older adults.

## Making Communities and Service Systems More Age-Friendly

De Biasi, Wolfe, Carmody, Fulmer, & Auerbach (2020) begin their discussion of an age-friendly public health system by noting that “programs that address older adult health continue to be siloed and under-resourced …. Programs such as Age-Friendly Communities, CDC’s Healthy Brain Initiative, Dementia-Friendly Communities, Age-Friendly Health Systems, and many other efforts are in nascent stages, operating independently.” To promote coordination, the Florida-based Age-Friendly Public Health Learning and Action Network linked 37 of Florida’s 67 county health departments, representing 65% of Florida’s older adult population, to build age-friendly health systems through a comprehensive *Framework for an Age-Friendly Public Health System*. For example, coordinated efforts from law enforcement, public works, parks and recreation, city planning, local businesses, health care systems, senior centers, and other community groups may be required to remove barriers to physical activity for older adults (and perhaps other age groups as well). The *Framework* will help build such a coordinated effort.

Evans, Oberlink, and Stafford (2020) developed a methodology for improving the aging-friendliness of communities. The authors examined outcomes from the AdvantAge Initiative, which was developed by the Visiting Nurse Service of New York to help communities develop aging-friendly policies. They examined results from AdvantAge in three communities: Memphis and Shelby County, Tennessee; New York City’s Chinatown neighborhood; and the state of Indiana. This community-engaged planning process prioritized accessible transportation, home modifications, and opportunities for socialization. These priorities differed by community, reflecting the particular resources and challenges of each community. To take one example, the community planning process in Shelby, TN led to three initiatives: Home Modifications for Low Income Seniors (Habitat for Humanity of Greater Memphis); No Hungry Senior (Metropolitan Inter-Faith Association); and the Coordinated Community Response to Elder Abuse (Family Safety Center of Memphis and Shelby County). The AdvantAge methodology is now widely used across highly diverse communities.

## Population Surveillance of Vulnerable Subgroups of the Aging Population

Taylor, Bouldin, Greenlund, and McGuire (2020) provide national estimates of subjective cognitive decline (SCD), the self-reported experience of worsening or more frequent confusion or memory loss. SCD is an easy-to-administer self-reported measure that signals disruption in the ability of someone to live independently and in some cases can be one of the earliest signs of Alzheimer’s disease. Analyzing data from the Behavioral Risk Factor Surveillances System, the authors find that more than 1 in 10 adults aged 45 years or older report SCD and that comorbid chronic conditions are more prevalent among those with SCD compared with those without SCD. They conclude that chronic condition self-care interventions delivered in community or health care settings should consider the importance of an individual’s cognitive status, including SCD.

The co-occurrence of cognitive impairment and chronic conditions, along with a lack of social supports, is also a concern among the growing number of older adults served by homeless housing programs. Yet existing screening tools used to determine priorities for admission to such settings do not adequately capture cognitive, medical, and psychosocial characteristics of older residents. Souza, Tsai, Pike, Martin, and McCurry (2020) collaborated with a leading housing services provider to develop and implement a systematic screening system for case managers to capture the cognitive, health, and psychosocial needs of formerly homeless older adults in these settings. Across four field sites they observed high prevalence of both objective and self-perceived cognitive problems suggestive of higher rates of cognitive impairment in these settings than is generally recognized. Their study demonstrates that it is feasible to implement a systematic intake system for case managers that identify cognitive, physical, and psychosocial needs of this vulnerable population.

Type 2 diabetes continues to be a public health priority in the United States. Physical activity has been identified as an effective strategy to manage Type 2 diabetes because it not only improves glucose tolerance but also lowers the risk of developing complications and related diseases. Nicklett and colleagues (2020) draw attention to whether physical activity levels, defined both in terms of rigor and frequency, change and are sustained following diagnosis with Type 2 diabetes. Using data from the Health and Retirement Study, they find a modest initial increase in physical activity after diagnosis that levels off, with those diagnosed at younger ages experiencing greater gains. Their findings highlight the potential for the delivery of a diagnosis of Type 2 diabetes as an important point of intervention for initiating behavior change, especially for older adults who may face barriers to physical activity, and the need for attention to sustaining improvements.

## Programmatic Innovations to Address Specific Needs Common Among Older Adults

What will it take to “chart a course for a dementia-prepared future?” Olivari, French, and McGuire (2020) outline progress in risk reduction and early detection of Alzheimer’s disease and related dementias. The public health infrastructure for dementia research, care, and policy has made clear strides between the initial CDC *Healthy Brain Initiative Road Map* release in 2007 and the *2018–2023 Road Map*, which “identifies 25 actions that state and local public health agencies and their partners can implement to promote cognitive health and address cognitive impairment and the needs of caregivers.” These involve changes in policy, systems, and environments. For example, changes in current public health campaigns can help destigmatize dementia. A social marketing effort by the Missouri Department of Health and Senior Services that included cognitive health in traditional public health messages (addressing, e.g., cardiovascular health, injury prevention, tobacco use, and nutrition and physical activity) reached about two thirds of local residents and led to increased use of the Alzheimer’s Association helpline and website. Other system changes extend public health protections to this vulnerable population. The Colorado Department of Public Health and Environment, for example, provides in-person training to first responders to help them identify wandering and abuse or neglect among people with cognitive impairment. The Minnesota Department of Health has an effort to mobilize community health workers to manage oral health among persons with dementia.

Disparities in dementia risk suggest a need for special efforts. The *Road Map for Indian Country* draws on tribal community strategies for addressing dementia and caregiving challenges. This integrated response to protecting cognitive health and extending supports to affected communities is a model for the changes in policy and environments required to prepare the United States for the growing prevalence of cognitive impairment expected to accompany an increasingly older population. Even with development of effective disease-modifying agents (still elusive after hundreds of failed clinical trials), we need public health if we are to realize the one-third reduction in dementia prevalence possible through risk reduction over the life span (Livingston et al., 2017). We also need public health to make our hospitals and emergency rooms, but also our airports and supermarkets, dementia friendly.

Mielenz and colleagues (2020) assessed an innovative person-centered wellness home for older adults. They delivered the Self-Management Resource Center Small Group Program, with and without wellness coaching, to older adults with chronic conditions in five South Bronx, NY public housing communities. Self-reported physical functioning improved in the group receiving coaching relative to the group participating in the standard wellness home model. This effort suggests the potential benefit of bringing services to public housing for chronic disease management. Other services that could be delivered on site include falls prevention, medication therapy review, assessment of vision and hearing, and many other programs with immediate benefit that do not require a clinical setting.

Finally, Mendez-Luck and colleagues (2020) assessed diabetes management programs among Latinx older adults compared to non-Latino white participants using the National Council on Aging’s large data repository of individuals who participated in diabetes self-management programs. They note that Latinos enrolled in workshops had a higher probability of completing at least four sessions than non-Latino whites, and also that Latinos enrolled in Spanish-language workshops (*Programa de Manejo Personal de la Diabetes*) had a higher probability of completing all six sessions. Notably, workshop location was less important for Latinx participation than language. These findings add to a growing knowledge base regarding implementation factors that affect effective delivery of aging services.

## Moving Forward

As this brief summary suggests, papers in the special issue met the charge of applying a public health perspective to aging subpopulations that span the continuum of health and vulnerability. Not all components of public health could be addressed, and undoubtedly many other kinds of aging could profitably be pulled into the conversation. Still, this collection brings to bear the tools of public health, and this approach forces us to think about aging more broadly than we ordinarily do. We look forward to a follow-up special issue, perhaps a few years on, where authors will move beyond programmatic efforts and health surveillance to population outcomes.

## References

[CIT0001] De Biasi A., Wolfe M., Carmody J., Fulmer T., & Auerbach J (2020). Creating an age-friendly public health system. Innovation in Aging, 4(1). doi: 10.1093/geroni/igz044 PMC7207260 32405542

[CIT0002] Evans L., Oberlink M., & Stafford P (2020). A practical methodology for improving the aging-friendliness of communities: Case studies from three U.S. communities. Innovation in Aging, 4(1). doi: 10.1093/geroni/igaa004 PMC7086477 32226824

[CIT0003] Livingston G., Sommerlad A., Orgeta V., Costafreda S. G., Huntley J., Ames D.,… Mukadam N (2017). Dementia prevention, intervention, and care. Lancet (London, England), 390, 2673–2734. doi:10.1016/S0140-6736(17)31363-6 28735855

[CIT0004] Mendez-Luck C., Govier D., Luck J., Julyan E., Mahakalanda S., & Herrera A (2020). Participation of Latinos in the diabetes self-management program & *Programa de Manejo Personal de la Diabetes*. Innovation in Aging, 4(1). doi: 10.1093/geroni/igaa006 PMC7078852 32206733

[CIT0005] Mielenz T. J., Tracy M., Jia H., Durbin L. L., Allegrante J. P., Arniella G., & Sorensen J. A (2020). Creation of the person-centered wellness home in older adults. Innovation in Aging, 4, igz055. doi:10.1093/geroni/igz055 31989045 PMC6974576

[CIT0006] Nicklett E. J., Chen J., Xiang X., Abrams L. R., Sonnega A. J., Johnson K. E.,… Assari S (2020). Associations between diagnosis with type 2 diabetes and changes in physical activity among middle-aged and older adults in the United States. Innovation in Aging, 4 (1). doi:10.1093/geroni/igz048 PMC7032072 32099903

[CIT0007] Olivari B. S., French M. E., & McGuire L. C (2020). The public health road map to respond to the growing dementia crisis. Innovation in Aging, 4(1). doi: 10.1093/geroni/igz043 PMC7207259 32405541

[CIT0008] Souza A. M., Tsai J. H., Pike K. C., Martin F., & McCurry S. M (2020). Cognition, health, and social support of formerly homeless older adults in permanent supportive housing. Innovation in Aging, 4(1). doi: 10.1093/geroni/igz049 PMC7207261 32405543

[CIT0009] Taylor C. A., Bouldin E. D., Greenlund K. J., & McGuire L. C (2020). Comorbid chronic conditions among older adults with subjective cognitive decline, United States, 2015-2017. Innovation in Aging, 4 (1). doi:10.1093/geroni/igz045 PMC6938465 31915725

[CIT0010] WHO (2015). *World report on ageing and health* Retrieved from https://www.who.int/ageing/events/world-report-2015-launch/en/

[CIT0011] WHO and National Institute of Aging (2011). *Global health and aging* Retrieved from https://www.who.int/ageing/publications/global_health.pdf

